# Adults with Treacher Collins Syndrome Share Comparable 3D Upper Airway Dimensions with Nonsyndromic Individuals

**DOI:** 10.1155/2024/6545790

**Published:** 2024-06-18

**Authors:** Renan Jhordan Mettelziefen dos Inocentes, Alexandre de Almeida Ribeiro, Maria Noel Marzano-Rodrigues, Marília Sayako Yatabe-Ioshida, Ivy Kiemle Trindade-Suedam

**Affiliations:** ^1^ Laboratory of Physiology Hospital for Rehabilitation of Craniofacial Anomalies University of São Paulo, São Paulo, Brazil; ^2^ School of Medicine Life and Health Science Institute Catholic University of Pelotas, Pelotas, Brazil; ^3^ Department of Orthodontics and Pediatric Dentistry School of Dentistry University of Michigan, Ann Arbor, MI, USA; ^4^ Laboratory of Physiology Hospital for Rehabilitation of Craniofacial Anomalies Bauru School of Dentistry University of São Paulo, Rua Silvio Marchione 3-20, Bauru—SP, CEP, São Paulo 17102-900, Brazil

## Abstract

**Purpose:**

Sleep apnea symptoms, such as snoring and daytime somnolence, are commonly observed in individuals with Treacher Collins Syndrome (TCS) and may be related to airway obstruction due to micro- and retro-gnathia. This study aims to three-dimensionally evaluate the upper airway using cone-beam computed tomography (CBCT) exams of adolescents (TCS-ADOL) and adults (TCS-ADUL) with TCS compared to a nonsyndromic group (CON).

**Materials and Methods:**

Twenty-six CBCT exams were divided into three groups: TCS-ADOL (*n* = 7) (13.14 ± 1.67 years): CBCT exams of TCS adolescents; TCS-ADUL (*n* = 10) (21.80 ± 4.39 years): CBCT exams of TCS adults; and CON (*n* = 9) (25.33 ± 8.57 years): CBCT exams of adult nonsyndromic individuals with Class II skeletal pattern. The variables analyzed were (1) total upper airway volume; (2) nasal cavity volume; (3) total pharyngeal volume; (4) nasopharyngeal volume; (5) oropharyngeal volume; (6) pharyngeal minimal cross-sectional area; (7) pharyngeal length; and (8) pharyngeal depth. Scans were analyzed by two examiners, and intra- and inter-rater agreement was calculated. A *p*-value of ≤0.05 was considered significant.

**Results:**

Although not statistically significant, the TCS-ADUL group showed decreased airway volume and minimal cross-sectional areas compared to the CON group. There were also significant differences between TCS-ADOL and TCS-ADUL, with significantly lower airway volumes in the TCS-ADOL group. Strong positive correlations were found between certain airway measurements in the TCS-ADOL group, which were not observed in adults.

**Conclusions:**

The upper airways of adults with TCS are dimensionally similar to those of nonsyndromic individuals, despite absolute value reductions found in the syndromic group. The reduced airway in the adolescent population suggests significant potential for growth, mainly in pharyngeal dimensions.

## 1. Introduction

**Figure 1:**
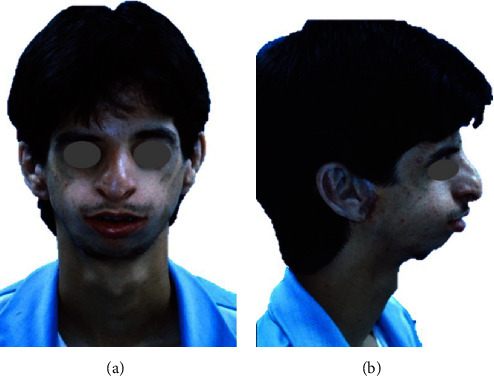
Clinical characteristics of facial anomalies in individuals with Treacher Collins Syndrome in frontal (a) and lateral (b) views. Note that the patient presents with a hypoplastic midface, a prominent nose, underdeveloped zygomatic bones, and micro- and retro-gnathia.

Patients with Treacher Collins Syndrome (TCS) present multiple facial and skeletal morphologic alterations such as hypoplastic midface, prominent nose, downward slanting eyes, inadequate development of the zygomatic bones, hypoplastic mandible, condylar abnormalities, among others. These features contribute to a severe convex profile, and, consequently, to a reduced upper airway (UA) [1, 2, 3, 4] (Figure 1).

Previous 3D studies suggest that patients with TCS present a decreased UA, mainly at the oropharyngeal level [3, 5]. Others suggest that the airway reduction can be multilevel [5, 6, 7]. These changes are believed to lead to airway problems, including obstructive sleep apnea (OSA) [8], characterized by multiple episodes of total (apnea), or partial (hypopnea) obstruction of the UA during sleep [9].

In combination with polysomnography exam (PSG), cone-beam computed tomography (CBCT) allows providers to better diagnose the structural deficiencies associated with OSA [10]. However, there are few available studies assessing the growth and development of the airway in adolescents and adults with TCS [3, 5, 6, 7] including pharyngeal length and depth, which are important variables related to OSA [11, 12, 13].

Therefore, the purpose of this study was to three-dimensionally assess the UA of patients with TCS. The null hypothesis was that the airway of patients with TCS (adolescents and adults) would be similar to that of nonsyndromic patients with skeletal Class II malocclusion.

## 2. Materials and Methods

This study was approved by the Institutional Review Board from HRAC/USP (Protocol number 15205413.7.0000.5441). For sample calculation, an alpha error of 5% and a beta error of 20% were considered. Adopting an expected standard deviation of 6 cm^3^ and significant difference between groups of at least 9 cm^3^ [9], an estimated sample size of 10 subjects per group was suggested.

The overall inclusion criteria were (1) full field of view (FOV); (2) presence of the galli crest at the upper limit of the image, and (3) presence of the third cervical vertebra at the lower limit of the image. For the TCS-ADUL group, additional inclusion criteria included a confirmed diagnosis of TCS and an age range of 18–50 years old. For the TCS-ADOL group: a confirmed diagnosis of TCS and an age range of 10–16 years old. For the control group, individuals were required to be between 18 and 50 years old and meet additional criteria, including being nonsyndromic, having a dolichofacial profile (FMA ≥ 30°), and having a Class II skeletal relationship (ANB ≥ 4). The exclusion criteria were a diagnosis of TCS with cleft palate, a history of previous surgeries related to the airway, and hypertrophic tonsils and/or adenoids [3, 5].

**Figure 2:**
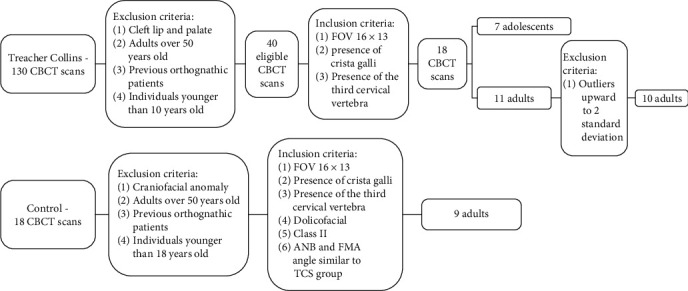
Flowcharts for CBCT scans selection. The upper flowchart refers to the TCS group while the lower flowchart refers to the control group.

From a total sample of 148 CBCT exams obtained retrospectively for diagnosis purposes, the sample for this study comprised 26 CBCT exams, divided into three groups as follows: TCS-ADOL (*n* = 7) (13.14 ± 1.67 years): CBCT exams of TCS adolescents; TCS-ADUL (*n* = 10) (21.80 ± 4.39 years): CBCT exams of TCS adults; and CON (*n* = 9) (25.33 ± 8.57 years): CBCT exams of adult nonsyndromic individuals with a Class II skeletal pattern (Figure 2). Tomographic scans of adults and adolescents with and without TCS were obtained from the records of HRAC/USP and from a private practice.

All images were obtained from i-CAT Next Generation Cone Beam imaging system (ISI-i-CAT Imaging System—cone beam, Next Generation i-CAT®, Imaging Sciences, Hatfield PA-USA) with the following specifications: field of view (FOV) 16 × 13 cm, exposure time 26.9 s, 120 kV, 37.07 mA and voxel size of 0.25 cm^3^. Images were exported as DICOM (Digital Imaging and Communications in Medicine).

**Figure 3:**
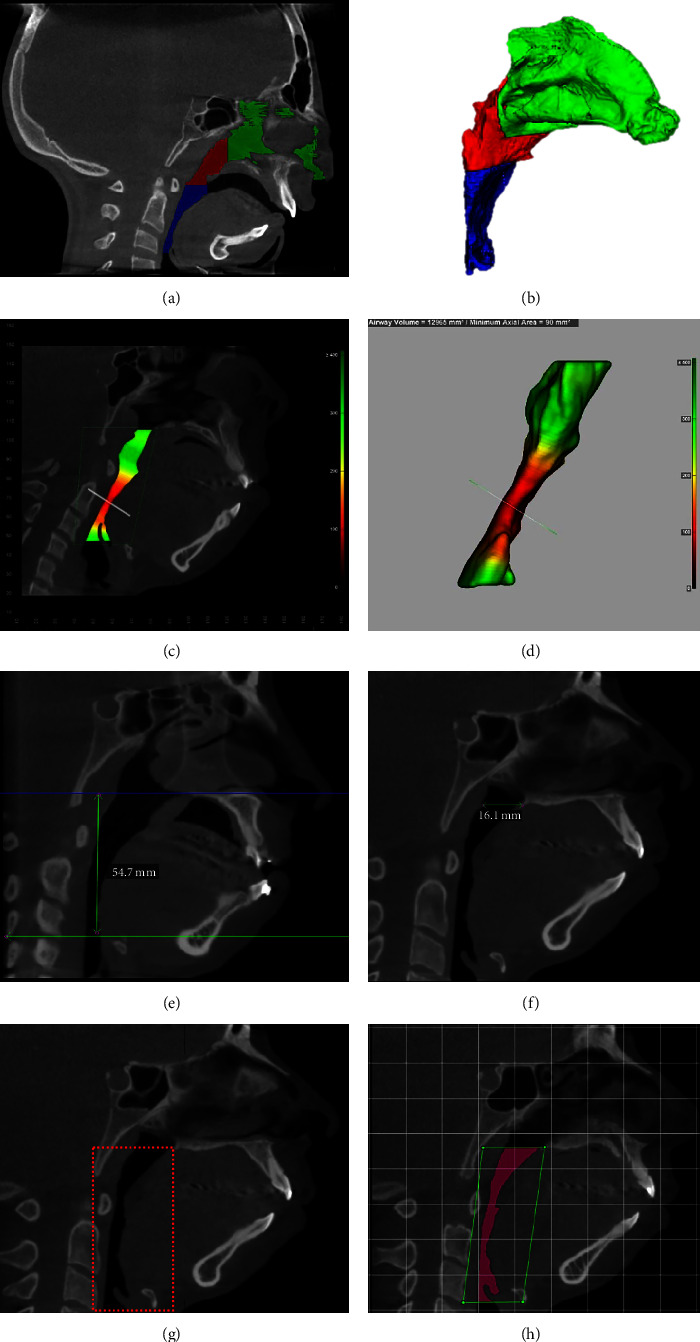
llustrative images of the delimitation areas for volumetric and linear measurements in individuals with Treacher Collins Syndrome. In images (a) (sagittal view) and (b) (3D), the green color represents the nasal cavity segmentation area, the red color represents the nasopharynx, and the blue color represents the oropharynx. In images (c) (sagittal view) and (d) (3D), the pharyngeal minimal cross-sectional area can be seen in the red pharyngeal area. Images (e) and (f) represent the pharyngeal length and pharyngeal depth acquisition, respectively. Images (g) and (h) represent the region of interest in ITK-SNAP and Dolphin Imaging, respectively.

Files were assessed using ITK-SNAP 3.8.0 software (http://www.itksnap.org, USA) [14, 15]. Three-dimensional segmentations of the regions of interest were assessed using a threshold between −1,000 and −400, values consistent with air density, which allows filling of the pharynx and nasal cavity [16]. To access the different volumes of the UA, the segmentation was divided into four distinct parts [15], as described in Table 1. The following variables were analyzed using ITK-SNAP (Figure 3): (1) volume of the total upper airway (TUP-VOL), which is represented by the sum of the three colors (green + red + blue); (2) volume of the nasal cavity (NC-VOL), represented by the green volume; (3) total pharyngeal volume (TP-VOL), represented by the sum of the red and blue colors; (4) nasopharyngeal volume (NP-VOL), represented by the red color; and (5) oropharyngeal volume (OP-VOL), represented by the blue color.

With Dolphin Imaging 11.8 software (Dolphin Imaging, Chatsworth, CA, USA) (Figure 3), variables analyses were (1) pharyngeal minimal cross-sectional area (P-mCSA), (2) pharyngeal length (P-Lenght), and (3) pharyngeal depth (P-Depth). The P-mCSA represents the minimal cross-sectional area perpendicular to a pharyngeal central line, which is automatically determined based on the segmentation of the pharynx.

The upper limit of the UAs was defined by a line parallel to the Frankfort plane that horizontally tangents the posterior nasal spine, and the lower limit by a line, also parallel to the Frankfort plane, that horizontally tangents the most anteroinferior point of the C3 vertebra [17], as seen in Figure 3. Likewise, P-Lenght was measured on Dolphin software using the same P-mCSA horizontal limits. In turn, P-Depth was measured from a line parallel to the Frankfort plane starting from the margin of the soft tissue adjacent to the nasopharynx toward the posterior wall of the pharynx.

The intraclass correlation coefficient (ICC) test was used to assess the intra- and inter-rater reproducibility [18]. Images were analyzed by two evaluators (RJMI and MNMR), previously trained to perform the measurements. Three months later, both evaluators repeated the measurements. The normal distribution of the variables was verified using the Shapiro–Wilk test, and intergroup comparisons of the quantitative variables V, mCSA, P-Length, and P-Depth were performed using the *t*-test for independent samples. Equality of variances was not assumed, and this was verified by the Levene test. The correlation between the variables was measured using the Pearson correlation test, which adopts the following score: *r* = 0–0.29 were considered as indicative of negligible correlation, *r* = 0.30–0.49 weak correlation, *r* = 50–69 moderate correlation, and *r* = 0.70–1.00 strong correlation [19].

## 3. Results

ICC values indicated a very good to excellent inter- and intra-rater agreement (ranging from 0.86 to 1.00), for all analyzed variables. Considering the high ICC values, the data obtained by evaluator 1 were used for presenting the results.

**Figure 4:**
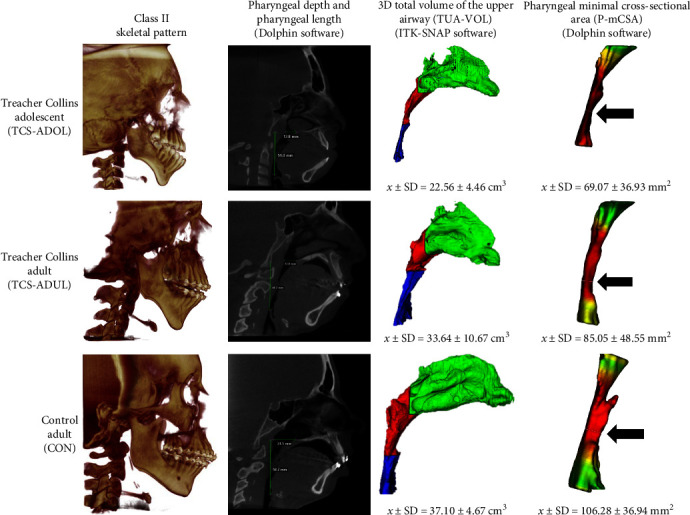
Three examples of patients from groups TCS-ADOL, TCS-ADUL, and CON showing 3D segmentation images of the Class II skeletal pattern, pharyngeal depth, pharyngeal length, 3D total volume of the upper airway, and pharyngeal minimal cross-sectional area.

Although not statistically significant, the TCS-ADUL group showed overall decreased volume (−9.3%) of the airways and its subdivisions, as well as decreased minimal cross-sectional areas (−19.9%) compared to the CON group. Similarly, decreased P-Length and P-Depth were observed in the TCS-ADUL group, although not statistically significant. No age differences were detected between the TCS-ADUL and CON groups (Figure 4). The mean values of the different variables assessed for the TCS-ADUL and CON groups are shown in Table 2.

When comparing the TCS-ADOL and TCS-ADUL groups, significantly lower volumes were observed for the TCS-ADOL group for the variables TUA-VOL, TP-VOL, NP-VOL, OP-VOL, and P-Length (Figure 4). NC-VOL, P-mCSA, and P-Depth were similar between the adolescent and adult groups (Figure 4). A significant difference was observed in the age of the two groups (TCS-ADOL and TCS-ADUL). The mean values of the different volumes measured between the TCS-ADOL and TCS-ADUL groups are also shown in Table 2.

**Figure 5:**
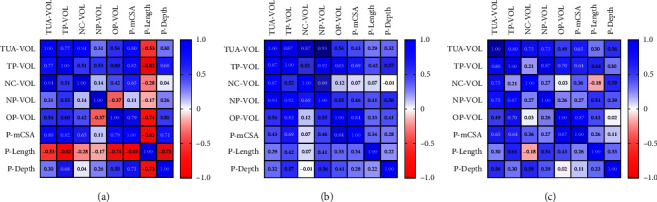
Pearson's *r* correlation matrix found in the three groups: positive correlations are shown in blue, and negative correlations are shown in red. The intensity of the color indicates the strength of the correlation. In (a), an adolescent individual with Treacher Collins Syndrome; in (b), an adult with Treacher Collins Syndrome; and in (c), a control individual.

In the TCS-ADOL group, strong positive correlations (>0.70) were observed between TUA-VOL and P-mCSA as well as TP-VOL × PmCSA. In the TCS-ADOL group, strong negative correlations were found between P-Length and TP-VOL, P-mCSA and P-Depth. In adults, with or without the syndrome, this negative pattern was not observed (Figure 5).

## 4. Discussion

The main result of the present study shows that even though not statistically significant, the total UA of adults with TCS is overall smaller compared to the control group (except OP-VOL). This is in accordance with a previous study from our research group, which has shown that the pharyngeal dimensions of individuals with TCS are negatively impacted by micro- and retro-gnathia, common characteristics of the syndrome, which may explain the increased prevalence of sleep-disordered breathing problems in this population [3, 5].

It is important to highlight that the control group was composed of severe skeletal Class II patients who required orthognathic surgery, and who naturally have smaller pharyngeal volumes and cross-sectional areas than skeletal Class I individuals [20]. The purpose of this selection was to tentatively reinforce the exclusive impact of the syndrome on the airway.

The hypothesis that the total UA of adolescent individuals with TCS is reduced in relation to adult individuals with TCS was confirmed. The nearly 50% increase in total airway volume in the TCS-ADUL group, compared to the group of adolescents with TCS, with statistical significance, validates the initial hypothesis. Thus, although the individuals evaluated in the two TCS groups are not the same, it can be inferred that the UAs increase in volume as the individual grows, by around 50% from 13 to 21 years of age. Our results align with those of Lin et al. [5], who assessed craniofacial and UA development in individuals with TCS, stating that the total UA volume of children with TCS is volumetrically smaller than that of individuals with TCS at skeletal maturity [5].

According to a study by Schendel et al. [21], as individuals grow, the total volume, lenght, and area of the airway increase until age 20, then remain relatively flat until age 50, when they all begin to decrease dramatically. The inclusion of individuals until 50 years is based on this evidence. Additionally, the nasal cavity volume reaches its greatest dimension between 15 and 18 years old [22].

These two studies support the age divisions used in our study. This is because our TCS-ADOL group has an age range between 11 and 15 years old, indicating that they are still in the growth phase [21, 22]. In contrast, the TCS-ADUL group has an age range between 18 and 31 years, suggesting that they have already completed UA volume growth. Although we did not evaluate the growth and development of the same group of individuals, we can assert, based on the cited articles, that there is approximately a 50% increase in total UA volume from adolescence to adulthood.

When assessing the different regions of airway separately, the volume of the nasal cavity did not show significant differences between TCS-ADUL and CON groups. However, it was 14.86% reduced in the TCS-ADUL group in comparison to the CON group, and even lower in the TCS-ADOL group (32% lower in comparison to TCS-ADUL group) (Table 2). Although previous studies from our group have shown that there is no association between internal nasal dimensions and severity of OSA [23], it is still understood that reduced nasal dimensions can predispose to OSA and this finding is of great importance since it may impact patient's quality of sleep [24].

Additionally, it is highlighted that studies have demonstrated a positive correlation between the volume of the NC-VOL and the retroposition of the maxilla [6, 7]. The authors demonstrated that hypoplasia of the middle third of the face, associated with malformation of the nasal and orbital bones, explains the reduced nasal volumes [6, 7].

The nasopharyngeal volume of the TCS-ADUL group also presented a reduction of 13% in relation to the CON group (Table 2), which can be explained as well by the shorter, hypoplastic, and retropositioned maxilla [2, 25]. This was a relevant clinical finding considering that this reduction can significantly negatively impact respiratory flow [26]. Applying Ohm's law to respiratory dynamics, it can be said that the greater the resistance of the UAs, in this case increased by reduced the volumes observed, the lower will be the airflow [27].

In the oropharyngeal region, the TCS-ADUL group presented volumes 73% greater than the TCS-ADOL group, with a statistical difference, indirectly evidencing the effect of growth on the dimensions of the UA (Table 2). In turn, the TCS-ADUL group had practically the same oropharyngeal volume as the control group, a fact already described and also observed in the dimensions of the NC-VOL and nasopharynx (Table 2).

The characterization of the areas of greatest airway constriction is essential for understanding respiratory dynamics. This is because very constricted areas make large volumes irrelevant, since, according to Poiseuille's Law, they can accelerate airflow and, consequently, increase intraluminal pressures, favoring pharyngeal collapse [13, 28].

In this regard, the P-mCSA reduction of almost 20% observed in the STC-ADUL group in relation to the CON group certainly has a great clinical impact, although we did not find a statistical difference (Table 2). As previously mentioned, this is because areas of great constriction can increase resistance to airflow [28]. Multiple factors, including the overall geometry of the UA, its curvature, the presence of hypertrophic tissues that also reduce the caliber of the UA, collaborate to determine the resistance and patency of the UA [29].

Additionally, the P-mCSA also increased by 23% from the TCS-ADOL to the TCS-ADUL (Table 2), indicating that the airway follows the individual's growth. In the evaluated sample, the mCSA was located in the oropharynx in all cases. This study corroborates with the findings of de Ribeiro et al. [3] in which the P-mCSA was also located in oropharyngeal region, and it was explained by the retropositioned mandible being associated with the location of the constriction [3]. The findings emphasize that micrognathia and the reduction in posterior facial height are closely related to the volumetric decrease of the UA [3].

A strong positive correlation (>0.70) between P-mCSA and pharyngeal volume in all groups (Figure 5), suggesting that reduced volumes imply areas of greater constriction, and the opposite is true. Therefore, interventions that result in an increased oro and nasopharynx, such as orthognathic surgeries for maxillomandibular advancement, may promote the expansion of mCSAs and improve the patency of the UA [15, 30].

Regarding P-Depth, it remained stable throughout growth in TCS groups. However, when compared to the CON group, the TCS-ADUL group was 18% smaller (Table 2). The authors believe that this is related to the maxillary underdevelopment, as well as the retrusion of the maxilla in relation to the skull base [6, 26].

On the other hand, the P-Length increased from the TCS-ADOL group to the TCS-ADUL group (Table 2), as expected, considering that the P-Length is influenced by growth and development [31]. However, when comparing the P-Length between the TCS-ADUL and CON group, no significant differences were observed (Table 2) which leads us to conclude that, even though the TCS group has a greater clockwise mandibular rotation [3], it did not affect its pharyngeal length. The P-Length seems to be of paramount importance due to its correlation to OSA, being considered a better predictor than the mCSA [11, 12, 32]. However, these data must be interpreted with care since our control group was composed predominantly of females (seven women, two men). According to Ronen et al. [33], the pharyngeal length is greater in males. Therefore, if there were more male individuals in the control group, this result could be different [33].

Interestingly, the TCS-ADOL group showed strong negative correlations between P-Length × TP-VOL, P-Length × OP-VOL, P-Length × P-mCSA, and P-Length × P-Depth (Figure 5), suggesting that the more elongated the pharynx is, the smaller its areas, volumes and depth. In adults, with or without the syndrome, this pattern was not observed. We must emphasize the difficulty of composing study groups of patients with syndromes who have undergone ionizing radiation exams. The craniofacial conditions analyzed here are rare. Additionally, there is the challenge of collecting tomographic scans retrospectively from patients without respiratory complaints and without previous surgeries.

Finally, when evaluating the data from the TCS-ADUL group and comparing it to the control group, it can be noted that this group of individuals has obstructions throughout their UA. A fact already described, and reinforced by our research group [3], by Plomp et al. [25] in their article.

Some of the challenges of this study refer to the control group: 78% of the control group was female, which *tends* to have a reduced airway [21, 22], a fact that could justify the absence of differences compared with the TCS-ADUL group. Another limitation was the lack of a skeletally balanced control group with a Class I malocclusion. However, obtaining tomographic images of these individuals would be unethical since there are no clinical reasons to do so.

The results of this study elucidate the sites of greatest reductions in the airways of individuals with TCS, for a better characterization of the predisposing factors for OSA. Considering that patients with craniofacial anomalies often present a reduced UA [3, 16, 34, 35, 36, 37, 38], it is expected a reduced and/or obstruction of the airflow, especially during sleep, when the pharyngeal muscle tone is reduced [28].

To the best of our knowledge, this is the first study to evaluate adults with Treacher Collins syndrome regarding total UA and nasal cavity volumes, as well as pharyngeal depth and length, using a skeletal Class II control group. Furthermore, we compared adolescents with Treacher Collins syndrome to adult subjects with the syndrome, all using cone-beam research.

However, the present study has some limitations, including the relative small sample size, the absence of a Class I control group, and the predominance of females in the control group. Future studies should include larger cohorts and/or different ethnicities and compare these results with polysomnography or polygraphy.

Additionally, future studies using computational fluid dynamics (CFD) should be conducted to better understand the association between the structure and function of the airway through computer simulations [39, 40]. Furthermore, studies assessing the occurrence of OSA in subjects with TCS are under development in our Sleep Lab and indicate that these individuals are at a higher risk for OSA compared to nonsyndromic subjects. This underscores the importance of identifying predisposing factors for OSA in the TCS population.

## 5. Conclusions

The airways of individuals with TCS are similar to those of nonsyndromic individuals, despite the absolute value reductions found in syndromic group. The reduced airway dimension in the adolescent population suggests a significant growth, particularly in pharyngeal dimensions.

## Figures and Tables

**Table 1:** Description of the delimitation area and limits used in the segmentation process.

Delimitation area	Delimitation limits
Total upper airway	Anterior limit—nostrils; inferior limit—most anterior inferior point of the C3 vertebra
Nasal cavity	Anterior limit—anterior limit of the total upper airway; posterior limit—most posterior and inferior point of the inferior nasal conchae
Nasopharynx	Anterior limit—posterior limit of the nasal cavity; inferior limit—most inferior point of the soft palate
Oropharynx	Superior limit—inferior limit of the nasopharynx; inferior limit—most inferior point of the total upper airway

**Table 2:** Dimensions of the upper airway and percent variation in individuals with Treacher Collins Syndrome and controls.

Variables	Groups						
	TCS-ADOL (*n* = 7)	TCS-ADUL (*n* = 10)	CON (*n* = 9)	TCS-ADUL vs. TCS-ADOL	TCS-ADUL vs. CON	TCS-ADUL vs. TCS-ADOL	TCS-ADUL vs. CON
	Mean (SD)	Mean (SD)	Mean (SD)	*p*-value	*p*-value	*Δ* %	*Δ* %
Years of age	13.14^a^ (1.67)	21.80^a^ (4.39)	25.33 (8.57)	0.00	0.28	—	—
Min–max	11–15	18–31	18–41	—	—	—	—
Gender	4♂, 3♀	6♂, 4♀	2♂, 7♀	—	—	—	—
Volumetric measures (cm^3^)							
Total upper airway	22.56^b^ (4.65)	33.64^b^ (10.67)	37.10 (4.67)	0.01	0.36	+49.11	−9.33
Nasal cavity	13.43 (3.32)	17.70 (6.14)	20.79 (2.85)	0.08	0.17	+31.79	−14.86
Total pharyngeal airway	9.13^c^ (1.74)	15.96^c^ (6.07)	16.31 (3.14)	0.00	0.87	+74.81	−2.15
Nasopharyngeal airway	5.52^d^ (1.50)	9.68^d^ (4.09)	11.16 (2.32)	0.01	0.34	+75.36	−13.26%
Oropharyngeal airway	3.62^e^ (1.59)	6.28^e^ (2.77)	5.15 (1.60)	0.02	0.28	+73.48	+21.94
Area measures (mm^2^)							
Oropharyngeal mCSA	69.07 (36.93)	85.05 (48.55)	106.28 (36.94)	0.45	0.29	+23.14	−19.98
Linear measures (mm)							
Pharyngeal length	52.90^f^ (5.79)	62.47^f^ (6.84)	63.56 (5.38)	0.00	0.70	+18.09	−1.71
Pharyngeal depth	17.06 (3.29)	16.44 (5.91)	20.19 (2.70)	0.78	0.0	−3.63	−18.57

TCS-ADOL, Treacher Collins Syndrome adolescents; TCS-ADUL, Treacher Collins Syndrome adults; CON, control group; mCSA, minimal cross-sectional area; *Δ* %, percent variation; vs, versus. Same letters indicate significant differences (*p* ≤ 0.05).

## Data Availability

This statement informs that data from the present study are not fully available to the scientific community due to the inclusion of sensitive information from volunteer patients. The restriction on access to the complete dataset is implemented to protect the privacy and confidentiality of study participants who shared highly personal and identifiable information. Despite recognizing the importance of scientific collaboration, the primary concern is ensuring the security of sensitive data. However, alternatives are being explored to share aggregated and anonymized findings that can contribute to the scientific community without compromising participant privacy. The statement expresses openness to discussing potential collaborations or alternative ways to facilitate knowledge exchange while respecting data confidentiality. Researchers interested in obtaining the original data (raw data) can contact the corresponding author directly at ivytrin@usp.br.
